# Reverse transcription recombinase-aided amplification assay for H5 subtype avian influenza virus

**DOI:** 10.1186/s12985-022-01807-0

**Published:** 2022-07-30

**Authors:** Suchun Wang, Yang Li, Fuyou Zhang, Nan Jiang, Qingye Zhuang, Guangyu Hou, Lijian Jiang, Jianmin Yu, Xiaohui Yu, Hualei Liu, Chenglong Zhao, Liping Yuan, Baoxu Huang, Kaicheng Wang

**Affiliations:** 1 China Animal Health and Epidemiology Center, 369 Nanjing Road, Qingdao, Shandong Province China; 2 Yanbian University, Agricultural College, Yanji, Jilin China; 3 Shandong Vocational Animal Science and Veterinary College, Weifang, China; 4 Key Laboratory of Animal Biosafety Risk Prevention and Control (South), Ministry of Agriculture and Rural Affairs, Qingdao, China

**Keywords:** Avian influenza, H5 subtype, Reverse transcription recombinase-aided amplification assay, Single temperature, Rapid, Sensitive

## Abstract

**Background:**

The H5 subtype avian influenza virus (AIV) has caused huge economic losses to the poultry industry and is a threat to human health. A rapid and simple test is needed to confirm infection in suspected cases during disease outbreaks.

**Methods:**

In this study, we developed a reverse transcription recombinase-aided amplification (RT-RAA) assay for the detection of H5 subtype AIV. Assays were performed at a single temperature (39 °C), and the results were obtained within 20 min.

**Results:**

The assay showed no cross-detection with Newcastle disease virus or infectious bronchitis virus. The analytical sensitivity was 10^3^ RNA copies/μL at a 95% confidence interval according to probit regression analysis, with 100% specificity. Compared with published reverse transcription quantitative real-time polymerase chain reaction assays, the κ value of the RT-RAA assay in 420 avian clinical samples was 0.983 (*p* < 0.001). The sensitivity for avian clinical sample detection was 97.26% (95% CI, 89.56–99.52%), and the specificity was 100% (95% CI, 98.64–100%).

**Conclusions:**

These results indicated that our RT-RAA assay may be a valuable tool for detecting H5 subtype AIV.

## Introduction

Influenza A virus (IAV), a member of the genus Influenza, family *Orthomyxoviridae,* contains eight single-stranded negative-sense RNA segments encoding at least 10 proteins [1, 2]. The IAV member avian influenza virus (AIV) can cause avian disease and public health problems. AIV can be classified into highly pathogenic avian influenza virus (HPAIV) and low pathogenic avian influenza virus (LPAIV) based on pathogenicity in chickens. The current HPAI H5N1 virus outbreak (from 2003 onwards) is, however, unprecedented in scale and geographic distribution, which have been prevalent among poultry in Asia, Europe and Africa. The viruses constantly undergo genetic drift and shift that permanently threatens the poultry industry and human health [3].

All LPAIV and HPAIV infections with H5 subtypes in poultry are notifiable to the World Organization for Animal Health [4]. Determination of the subtypes of AIV is of importance for the diagnosis of these infections, which can be achieved by analysis of the amino acid sequence of the cleavage site in the AIV hemagglutinin (HA) gene [5]. Besides, there are many methods, such as restriction enzyme cleavage patterns, probe hybridization and real time reverse transcription quantitative polymerase chain reaction (RT-qPCR) [5–8]. Based on the widespread availability of RT-qPCR technology in diagnostic laboratories, it has been used to identify H5 subtype HPAIV of the goose/Guangdong (gs/GD) lineage.

Recently, rapid isothermal amplification techniques have been developed, such as loop-mediated isothermal amplification (LAMP) [7], recombinase polymerase amplification (RPA) [8], recombinase-aided amplification (RAA) and strand displacement amplification (SDA) [9], and widely used in clinical detection. Among these rapid nucleic acid detection methods, reverse transcription recombinase-aided amplification (RT-RAA) is a rapid thermostatic nucleic acid amplification technology that utilizes a recombinant enzyme obtained from bacteria or fungi. At room temperature, the recombinant enzyme can tightly bind to the primer DNA to form polymer. When the primer recognizes the template DNA for a complementary sequence that perfectly matches it, with the help of a single-stranded DNA binding protein, it opens the double-stranded structure of the template DNA under the action of DNA polymerase, a new cDNA strand is formed, and the amplification product grows exponentially [10]. This technology has the characteristics of high sensitivity, stronger specificity and reliability.

In order to quickly and simply detect H5 subtype AIV and provide more sufficient preparation time for prevention and control, an RT-RAA assay was designed and its analytical specificity and sensitivity were evaluated in this study. The result suggested that RT-RAA assay meet the need of field testing, and presented a rapid and sensitive detection method that could be used as an alternative to animal inoculation or nucleotide sequencing.

## Materials and methods

### Ethics statement

This study was conducted according to the animal welfare guidelines of the World Organization for Animal Health and was approved by the Animal Welfare Committee of the China Animal Health and Epidemiology Center (CAHEC). CAHEC has permission to study the activities of HPAIV. The swab samples were collected after being granted permission by several relevant parties, including the Ministry of Agriculture and Rural Affairs of China, CAHEC, the relevant veterinary sections of the provincial and national governments, and the relevant farm owners.

### Samples and extraction of viral nucleic acids

All the nucleic acid of AIVs, Newcastle disease virus (NDV) and infectious bronchitis virus (IBV) used in this study were identified in our laboratory (the National Avian Influenza Professional Laboratory in China Animal Health and Epidemiology Center) and were stored at − 80 °C. The samples were centrifuged at 12,000 × *g* for 10 min and the supernatant from each sample was used for RNA extraction on the QIAxtractor platform using a cador Pathogen 96 QIAcube HT kit (Qiagen). The extracted RNA was stored at − 80 °C for subsequent tests.

### Preparation of viral RNA standard

An H5 subtype AIV viral RNA standard was developed using the reference strain A/duck/Yunnan/5310/2006(H5N1) (GenBank accession number CY030889). A 1776-bp fragment of the whole HA gene of H5 subtype AIV was constructed and digested with enzyme. The digested plasmid was purified with DNA recovery kit (Thermo Fisher Scientific) and transcribed in vitro with RNA Production System-T7 kit. The products were extracted and purified by Rneasy MiNi Kit to remove the heteroproteins and various ions in the system. The concentration of cRNA standard transcribed in vitro was accurately measured by Nano Drop nucleic acid quantitative analyzer, and the copy number was calculated. The cRNA standard was continuously diluted with RNase free H_2_O in tenfold gradient. The sensitivity of the RT-RAA was evaluated by real-time fluorescence detection, using the diluted cRNA standard ranging from 10^6^ to 10^1^ copies/μL.

### Design of H5 RT-RAA primers and exo-probes

To detect H5 subtype AIV, a total of 4636 available HA gene sequences of H5 subtype AIV obtained from GenBank database were aligned, which contained the HA gene sequence of all currently circulating branches of H5 subtype AIV, and highly conserved regions were subsequently identified with Molecular Evolutionary Genetics Analysis (MEGA) software 6.0 [11] for the design the gene-specific primers and probes. Primers were designed using OLIGO 7 software [12] and showed no major nonspecific sequence similarities by BLAST analysis. Three H5 forward primers and five reverse primers were designed to select the best primers and probes in combination. The appropriate primers and probe combination were selected by sequence analysis, which were shown in Table 1. The 30th base at the 5′ end of the probe was labeled with the FAM fluorophore. The 30th base was connected to the abasic site tetrahydrofuran (THF). The 31st base was labeled with the BHQ1 quencher, and the 3′ end was modified by 3′ block. All the primers and probes were synthesized by Sangon Biotech.

**Table 1:** Primer and probe sequences used for RT-RAA and RT-qPCR assays

Primer	Sequence (5′–3′)	Size (bp)	Gene	Source
H5-F	CAGTTTGAGGCYGTTGGAAGGGAATTTAAYAA	32	HA	This study
H5-R	CTTGTCRTAAAGGTTCTTGACATTTGAGTCAT	32	HA	This study
H5-P	CTAGATGTCTGGACTTATAATGCTGAACT/i6FAMdT//THF//iBHQ1dT/GGTTCTCATGGAAAAT[C3-spacer]	47	HA	This study
H5 + 1456	ACGTATGACTATCCACAATACTCAG	25	HA	[13]
H5-1685	AGACCAGCTACCATGATTGC	20	HA	[13]
H5 + 1637	FAM-TCAACAGTGGCGAGTTCCCTAGCA-TAMRA	24	HA	[13]

### RT-RAA for detection of H5 subtype AIV

The appropriate primers and exo-probes were screened, and the RT-RAA reaction was performed with an RT exo kit in 50 μL reaction mixture including the necessary enzymes and reagents for RT and DNA amplification in lyophilized pellets (Jiangsu Qitian Bio-Tech Co. Ltd.). The reaction mixture contained the following: 2 μL RNA template, 25 μL rehydration buffer, 15.7 μL deionized distilled water, 2.5 μL magnesium acetate, 2.1 μL each primer (10 μM) and 0.6 μL target-specific RT-RAA exo-probe (the probe concentration is 40 ng/μL). For amplification, the tubes were then transferred to a tube holder in an RT-RAA fluorescence detection device (QT-RAA-F7200; Jiangsu Qitian Bio-Tech Co. Ltd.) set at 39 °C for 20 min. Each run included nuclease-free water as a negative control. The results of the RT-RAA interpretation criteria were determined by the slope of amplification curve of the RT-RAA fluorescence detection device (QT-RAA-F7200), the test result is positive when the slope k value was greater than or equal to 20, in addition, the test result is negative.

### Analytical sensitivity and analytical specificity of RT-RAA

Recombination plasmid of HA gene of H5 subtype has been constructed using the pEASY™-T5 Zero Cloning Kit. The plasmid was cleaved using a suitable restriction enzyme. The digested plasmid was purified with a DNA recovery kit, the concentration was measured with a Nano Drop nucleic acid quantifier, and then an appropriate amount was taken as a template according to its concentration, and the RNA Production System-T7 kit was used for in vitro transcription. The in vitro transcription products were extracted and purified with the Rneasy MiNi Kit to remove impurities and various ions in the system. Serial tenfold dilutions of known concentrations of in vitro transcribed cRNA standards with RNase Free H_2_O.

A dilution range of H5 subtype HA plasmid standard was used to select the appropriate RAA primers and exo-probe combination. The selected appropriate primers and exo-probes were verified by H5 RT-RAA analysis. To determine the analytical sensitivity of the H5 RT-RAA assay, we detected the H5 subtype AIV molecular RNA standard over a dilution range of 10^7^–10^1^ copies/μL under the optimal RT-RAA conditions, with eight replicates for each dilution.

The analytical specificity assay of the RT-RAA for H5 subtype AIV was evaluated using four H5-positive AIVs (one strain in clade 2.3.2.1, one in clade 7, and 55 in clade 2.3.4.4), 10 other subtype AIVs (H1N2, H3N2, H4N2, H6N2, H7N3, H7N9, H9N2, H10N7, H11N9), two NDVs and two IBVs. These viruses are the main respiratory viruses affecting birds and were previously identified by our laboratory. The details of all the viruses tested are listed in Table 2.

**Table 2:** Some information about the samples used for analytical sensitivity and analytical specificity assay in the study

Sample	Virus	HA subtype(clade)	H5 RT-RAA assay	H5 RT-qPCR assay
K144	AIV	H5N1(2.3.2.1)	+	+
QD1	AIV	H5N2(7)	+	+
G2324	AIV	H5N6(2.3.4.4)	+	+
G2084	AIV	H5N6(2.3.4.4)	+	+
Q221	AIV	H1N2	−	−
X1330	AIV	H3N2	−	−
P174	AIV	H4N2	−	−
A1267	AIV	H6N2	−	−
H7N3	AIV	H7N3	−	−
1605	AIV	H7N9	−	−
X169	AIV	H9N2	−	−
H9	AIV	H9N2	−	−
T55	AIV	H10N2	−	−
S82	AIV	H11N2	−	−
ND	NDV	/	−	−
JS1816	NDV	/	−	−
M41	IBV	/	−	−
H52	IBV	/	−	−

### Detection and evaluation of clinical samples by H5 RT-RAA

To test the efficacy of the method for clinical application, 420 throat and cloacal swabs and tissue samples whose hosts contained chickens, ducks, geese, pigeons and wild birds were collected from 29 live poultry markets and farms in six provinces. Throat and cloacal swabs were immediately placed in 1 mL antibiotic-containing PBS as described above and then stored at − 80 °C until total nucleic acids were extracted with the above viral RNA extraction kit [14]. The tissue samples were sheared and ground after adding PBS, and their supernatants are extracted after centrifugation for nucleic acid extraction. We evaluated the performance of the RT-RAA assay in 420 avian clinical samples and compared it with the published RT-qPCR method for H5 subtype AIV. The primers and probe of the RT-qPCR assay are listed in Table 1 [13]. The RT-qPCR assay was conducted with the One Step PrimeScript RT-PCR Kit (Takara).

### Statistical analysis

To determine the RT-RAA detection limit, a probit analysis was performed at a 95% confidence interval (CI), and the κ and *p* values of RT-qPCR and RT-RAA were calculated [15]. In addition, we calculated the sensitivity and specificity of RT-qPCR and RT-RAA for detection in clinical samples of poultry. All statistical analyses were performed in SPSS 21.0 (IBM).

## Results

### Analytical sensitivity of RT-RAA

**Fig. 1:**
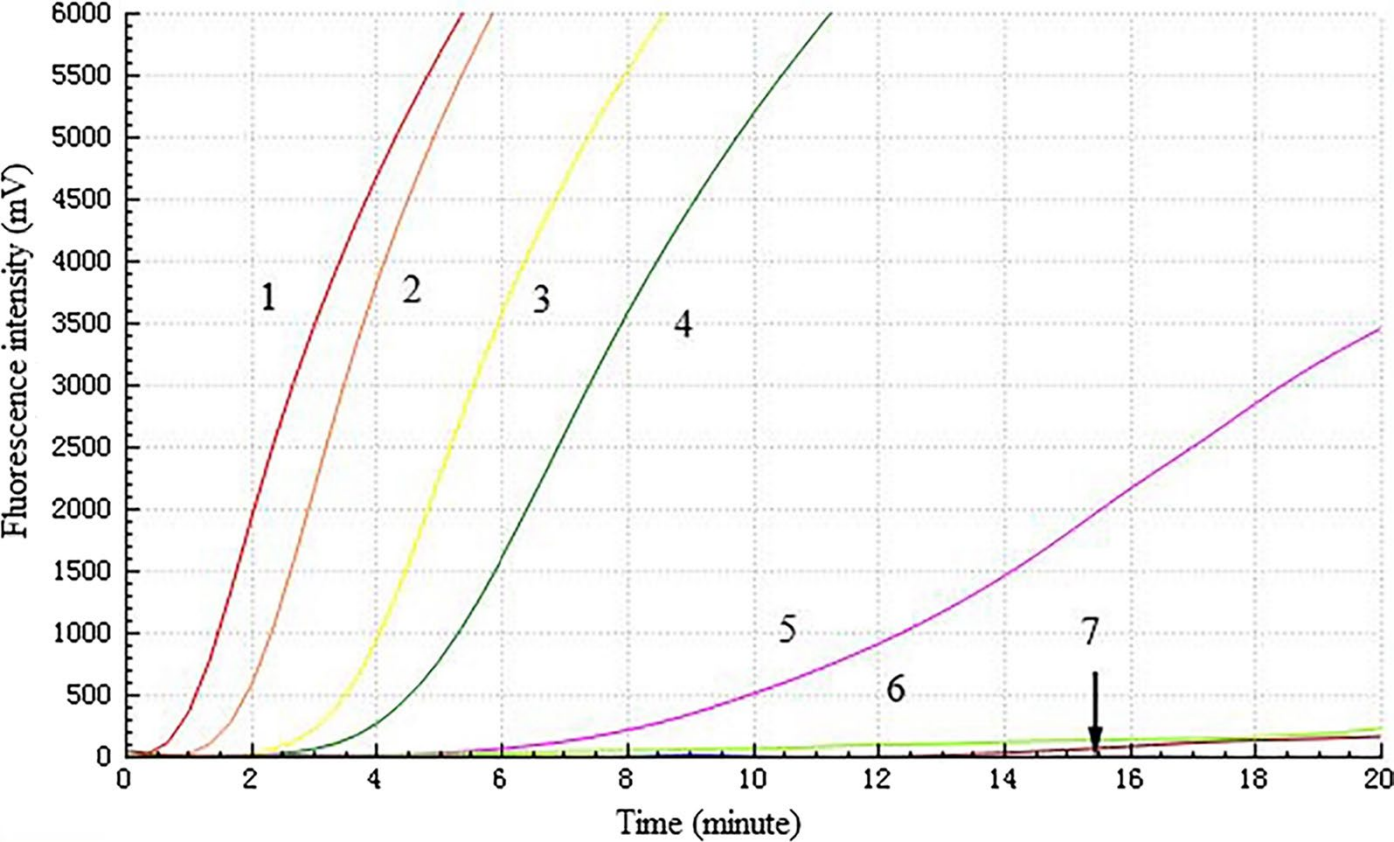
Analytical sensitivity of the H5 RT-RAA assay. A dilution range from 10^6^ to 10^1^ copies/μL of H5 subtype AIV cRNA was used to evaluate the detection limit of H5 RT-RAA assay. Negative represents negative control. 1–6: 10^6^ copies/μL–10^1^ copies/μL; 7: negative control

The sequences of the appropriate H5 RT-RAA primers and exo-probe are listed in Table 1. The detection results of RT-RAA sensitivity assay are shown in Fig. 1. The primer and probe combinations designed by the present invention have a cRNA concentration of 10^6^ copies/μL, 10^5^ copies/μL, 10^4^ copies/μL, and 10^3^ copies/μL, 10^2^ copies/μL, 10^1^ copies/μL, a fluorescence amplification curve appears. Therefore, the detection limit of H5 RT-RAA assay was 10^2^ cRNA copies/μL (Table 3, Fig. 1).

**Table 3:** Assay data used for probit analysis to calculate the detection limits of H5 RT-RAA

Copies/μL	No. of positive samples/No. of samples tested by the RT-RAA assays for detection of H5 subtype AIV^a^
10^6^	8/8
10^5^	8/8
10^4^	8/8
10^3^	5/8
10^2^	2/8
10^1^	0/8

^a^Each dilution was tested in a total of eight replicates

### Analytical specificity of RT-RAA

**Fig. 2:**
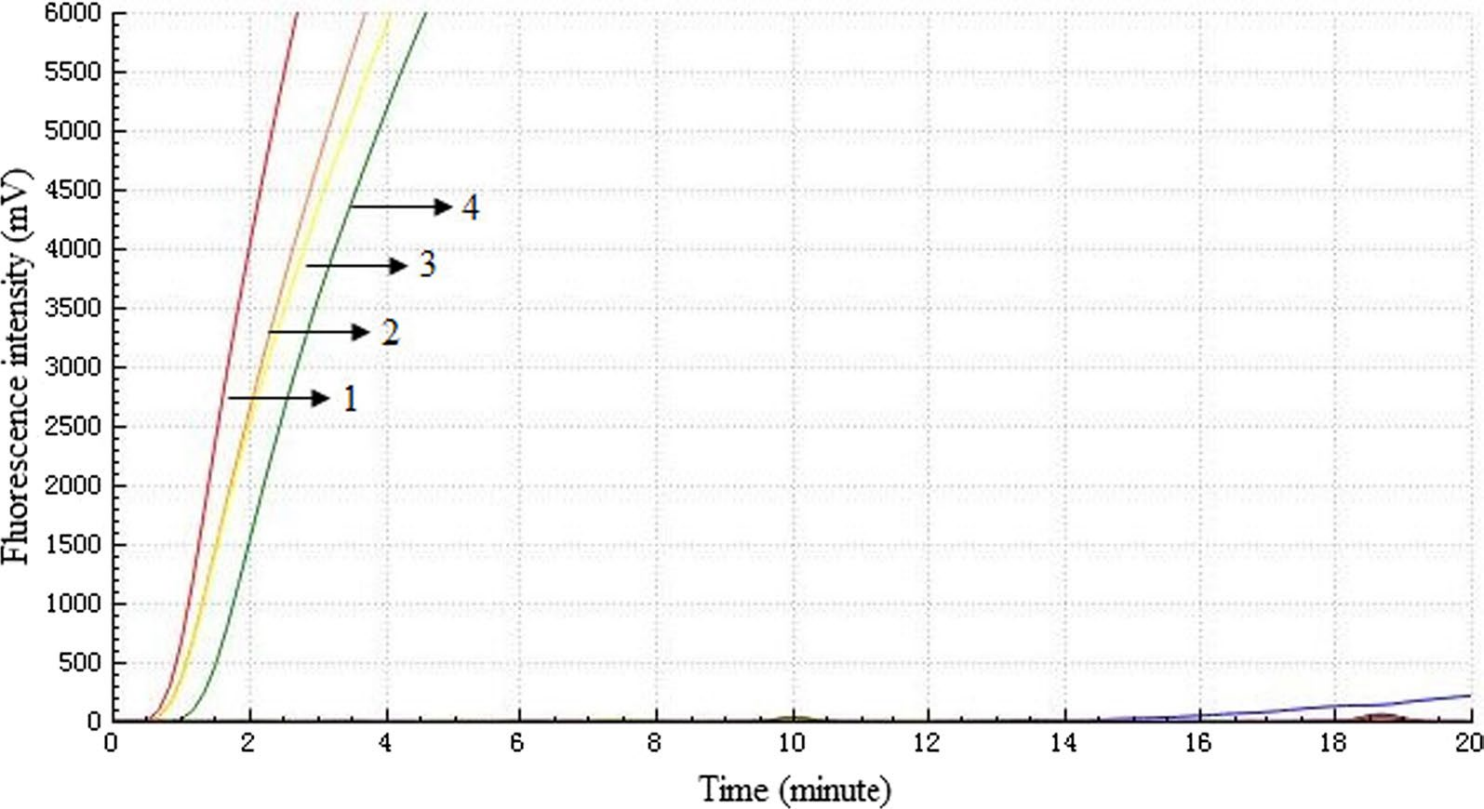
Analytical specificity of the H5 RT-RAA assay. Detection signals were recorded by real-time fluorescence RT-RAA with four samples including H5 subtype AIVs (H5N1, H5N2 and H5N6), while no signals were detected from the 14 samples including other subtype AIVs, NDVs, IBVs and negative controls. 1: K144 (H5N1); 2: QD1 (H5N2); 3: G2324 (H5N6); 4: G2084 (H5N6)

The test group corresponding to the H5 subtype AIV RNA template showed standard fluorescence detection curves, and the other virus test groups and negative control groups did not show amplification curves. Thus, the RT-RAA assay did not crossreact with other subtype AIVs, NDVs and IBVs and demonstrated high specificity for the detection of H5 subtype AIV (Fig. 2).

### Evaluation of the RT-RAA for clinical samples

The 420 conserved avian clinical samples collected from live poultry markets were tested by RT-RAA assay and compared with RT-qPCR. A threshold cycle value of 36 was used as the cut-off for a positive result in RT-qPCR. RT-qPCR detected 73 of the 420 samples as positive (17.38%), while RT-RAA correctly identified and differentiated 71 positive samples, with a sensitivity of 97.26% (95% CI, 89.56–99.52%) and 100% specificity (95% CI, 98.64–100%) (Table 4). The κ value for RT-RAA and RT-qPCR was 0.983 (*p* < 0.001).

**Table 4:** Detection of H5 subtype AIV in avian clinical samples

		RT-RAA		Total	Kappa (κ)	*p* value of kappa	Sensitivity% (95% CI)	Specificity% (95% CI)
		Positive	Negative					
RT-qPCR	Positive	71	2	73	0.983	< 0.001	97.26 (89.56–99.52)	100 (98.64–100)
	Negative	0	347	347				
Total		71	349	420				

## Discussion

The frequent variation of AIV antigen increases the difficulty of AIV detection. Among all the AIV subtypes, H5 subtype HPAIV often leads to high morbidity and mortality in poultry. Nowadays, the H5 subtype AIV is widely prevalent and of significant concern to the poultry industry and public health in China. Therefore, early detection of AIV and H5 subtype is necessary in the surveillance and control of AIV outbreaks. Until now, there have been many methods used in AIV detection, such as gold immunochromatographic assay [16], microarray [17], immunosensor [18], immunofluorescence [19] and enzyme-linked immunosorbent assay [20]. However, many methods require complex and costly devices and are difficult to perform in the field. So far, many detection methods have been used for rapid detection of the AIV in the field, such as nucleic acid sequence amplification [21], RT-LAMP and RT-RPA [22]. Moreover, as the mutation rate of the H5 subtype AIV accelerates, the previous detection methods may not meet the actual detection needs. Some of them fail to subtype AIV, while others cannot be applied in early diagnosis owing to inadequate sensitivity [23], so a rapid diagnostic method capable of detecting all avian influenza epidemic strains is needed.

RAA is a novel isothermal amplification and detection assay requiring only 20 min to complete, while RT-qPCR and conventional PCR need longer time. In PCR, thermocycling is required for double-stranded DNA separation, primer binding and amplification. RT-RAA could produce a positive signal in as little as 4 min and be completed in 30 min, at half the cost of RT-qPCR. RAA can be carried out using a portable device with no complicated processes, while the instruments for RT-qPCR and conventional PCR are more expensive. RAA can also use reverse transcriptase and a fluorescent probe system to detect RNA amplicons in real time [17]. Until now, RAA has widely been used for detecting human pathogens, including *Salmonella*, *Listeria monocytogenes*, respiratory syncytial virus, coxsackievirus, MERS-CoV, SARS-CoV-2, *Mycobacterium Tuberculosis*, hepatitis B virus and *Schistosoma japonicum*-specific gene fragments and there have been no previous reports of the use of RAA for detecting H5 subtype AIV [24–31]. RAA has also been used in the detection of *Staphylococcus aureus* in milk, African swine fever virus and animal derived ingredients in exported food, such as pigs, cows and chickens [32–36]. However, compared with fluorescence and ordinary PCR, the cost of RT-RAA is relatively high at present.

In this study, an H5 RT-RAA assay was created, which indicated the potential value of our method for early detection and rapid diagnosis of the H5 subtype AIV. In the detection of experimental and clinical samples, this method showed higher sensitivity along with high efficiency. RT-qPCR detected 73 of the 420 samples as positive (17.38%), while RT-RAA correctly identified 71 positive samples, with a sensitivity of 97.26% (95% CI, 89.56–99.52%) and 100% specificity (95% CI, 98.64–100%) (Table 4). The κ value for RT-RAA and RT-qPCR was 0.983 (*p* < 0.001). Moreover, the clinical swab samples detected as positive in RT-RAA were also certified as positive by PCR detection and sequencing. According to the primer/exo-probe design method and the sample detection results, the RT-RAA method was considered to be suitable for detecting almost all H5 subtypes. To the best of our knowledge, this is the first RT-RAA method for the detection of multiple H5 clades in AIV.

In conclusion, the RT-RAA method established in our study quickly and accurately identified H5 subtype AIV, including the current epidemic strains, which meets the need for H5 subtype AIV testing. This RT-RAA is expected to detect emerging H5 subtype AIV rapidly and provide a powerful and valuable tool for the control of H5 subtype AIV.

## Data Availability

All data generated or analyzed during this study are included in this published article [and its supplementary information files].
